# Incremental Value of Immediate Postpartum POCUS for Risk Stratification of Adverse Maternal Outcomes in Hypertensive Disorders of Pregnancy

**DOI:** 10.3390/jcm15134989

**Published:** 2026-06-26

**Authors:** Meijing Zhao, Shijie Zhang, Huilan Hong, Guorong Lyu

**Affiliations:** 1 Department of Obstetrics, Second Affiliated Hospital of Fujian Medical University, Quanzhou 362000, China; m18900329265@163.com (M.Z.); hhl831911@fjmu.edu.cn (H.H.); 2 Department of Ultrasound, Second Affiliated Hospital of Fujian Medical University, Quanzhou 362000, China; 1230920139@fjmu.edu.cn; 3 Department of Clinical Medicine, Quanzhou Medical College, Quanzhou 362000, China

**Keywords:** maternal hemodynamics, pulmonary edema, cardiac function, risk stratification

## Abstract

**Objective**: To evaluate the incremental value of immediate postpartum point-of-care ultrasound (POCUS) parameters for risk stratification of adverse maternal outcomes (AMO) in women with hypertensive disorders of pregnancy (HDP). **Methods**: This prospective observational cohort study was conducted between January 2024 and March 2025 in the Labor Ward of the Second Affiliated Hospital of Fujian Medical University. Women diagnosed with HDP after 20 weeks of gestation underwent standardized lung and cardiac POCUS examinations within 2 h after delivery. Maternal demographic, laboratory, and ultrasound variables were compared between women with and without AMO during the 42-day postpartum follow-up period. A baseline clinical model was constructed using conventional clinical and laboratory variables. Ultrasound parameters were subsequently added individually to assess their incremental value for risk stratification. Model performance was evaluated using the Brier score, Akaike Information Criterion (AIC), and area under the receiver operating characteristic curve (AUC). **Results**: A total of 160 women were included, of whom 35 (21.88%) experienced AMO. Compared with women without AMO, those with AMO showed significantly higher echo comet score (ECS), left atrial volume index (LAVI), and left ventricular index of myocardial performance (LIMP), while left ventricular E/A ratio was significantly lower (all *p* < 0.05). The baseline clinical model yielded an AUC of 0.88. Addition of ECS, LAVI, or LIMP individually improved model discrimination, with corresponding AUCs increasing to 0.93. These ultrasound-enhanced models also demonstrated lower Brier scores and AIC values compared with the baseline clinical model alone. **Conclusions**: In women with HDP, immediate postpartum lung and cardiac ultrasound parameters differed significantly according to postpartum outcome status. Incorporation of ultrasound-derived maternal hemodynamic indicators provided incremental value beyond conventional clinical variables for risk stratification of AMO. Immediate postpartum POCUS may therefore serve as a practical bedside adjunct for early identification of high-risk women with HDP and may help guide individualized postpartum monitoring. To our knowledge, this is the first prospective study evaluating the incremental value of immediate postpartum lung and cardiac POCUS for risk stratification in women with HDP.

## 1. Introduction

Hypertensive disorders of pregnancy (HDP) remain a major cause of maternal morbidity and mortality worldwide [1]. Women with HDP are at increased risk of adverse maternal outcomes (AMO), including pulmonary edema, cerebrovascular events, renal dysfunction, hepatic injury, and postpartum hemorrhage [2,3,4,5]. Optimal hemodynamic management is crucial in this population. Despite advances in obstetric management, early identification of women at high risk of deterioration remains clinically challenging [6,7,8].

The intrapartum and immediate postpartum periods are characterized by profound hemodynamic changes, including increased preload and cardiac output resulting from uterine contractions, autotransfusion, and fluid redistribution [9,10,11,12]. In women with HDP, these physiologic changes may further aggravate endothelial dysfunction, impaired vascular compliance, and subclinical cardiac dysfunction, thereby increasing the risk of maternal decompensation [13,14]. Importantly, conventional clinical and laboratory indicators provide only limited insight into these dynamic hemodynamic alterations during labor and the early postpartum period.

Point-of-care ultrasound (POCUS) has emerged as a rapid and noninvasive bedside tool for hemodynamic assessment in critical care and emergency medicine [15,16,17]. Lung ultrasound enables semiquantitative assessment of extravascular lung water and early detection of pulmonary congestion [18,19,20]. Focused cardiac ultrasound can provide real-time evaluation of cardiac structure, filling status, and ventricular performance. Together, lung and cardiac ultrasound may offer a more comprehensive assessment of maternal cardiovascular adaptation during the immediate postpartum period than conventional clinical assessment alone [21,22,23,24].

Several prognostic models for HDP, including the fullPIERS and PREP models, primarily rely on demographic, clinical, and laboratory variables [4,5,25,26]. Although these models have demonstrated acceptable predictive performance, they do not incorporate direct assessment of maternal cardiopulmonary adaptation during the immediate postpartum period. Consequently, important hemodynamic abnormalities may remain undetected despite apparently stable clinical findings. Whether immediate postpartum ultrasound-derived indicators provide incremental value beyond conventional clinical variables for risk stratification in women with HDP remains unclear.

Therefore, this prospective observational study aimed to evaluate the association between immediate postpartum lung and cardiac ultrasound findings and postpartum AMO in women with HDP and to determine whether incorporation of ultrasound-derived maternal hemodynamic parameters could improve risk stratification beyond conventional clinical and laboratory indicators.

## 2. Materials and Methods

### 2.1. Study Design and Participants

**Figure 1:**
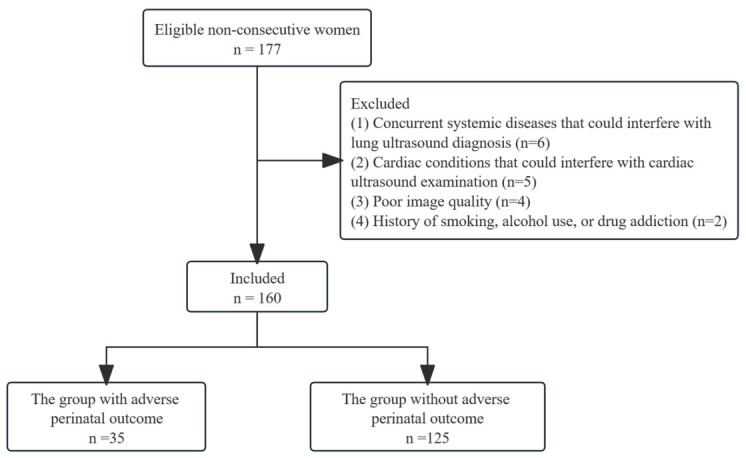
Flow diagram for inclusion and exclusion.

This prospective observational cohort study was conducted in the Labor Ward of the Second Affiliated Hospital of Fujian Medical University between January 2024 and March 2025. During the study period, 189 women with HDP were eligible at our center. Among them, 177 were enrolled (daytime cases), and 160 eligible participants were finally included in the analysis. Thus, the enrolled cohort accounted for 93.7% of eligible HDP patients, and the final analytic cohort accounted for 84.7% of eligible HDP patients during the study period (Figure 1).

Eligible participants were women aged ≥18 years who met the diagnostic criteria for HDP (systolic blood pressure ≥ 140 mmHg and/or diastolic blood pressure ≥ 90 mmHg, measured at least 4 h apart after 20 weeks of gestation in previously normotensive women) and were able to provide written informed consent. HDP was defined according to the American College of Obstetricians and Gynecologists (ACOG) Practice Bulletin criteria. Participants were further classified as having gestational hypertension, preeclampsia, preeclampsia with severe features, eclampsia, chronic hypertension with superimposed preeclampsia, or chronic hypertension complicating pregnancy [27,28].

Exclusion criteria included (1) systemic diseases potentially affecting lung ultrasound interpretation, including pneumonia or interstitial lung disease; (2) structural or functional cardiac disorders potentially interfering with echocardiographic assessment; (3) inadequate image quality; and (4) history of smoking, alcohol abuse, or drug addiction.

The study protocol was approved by the Medical Research Ethics Committee of the Second Affiliated Hospital of Fujian Medical University (Approval No. 2022FYLLSZ278; 30 August 2022). Written informed consent was obtained from all participants.

### 2.2. Clinical and Laboratory Variables

Prospectively recorded maternal variables included age, gestational age, height, prenatal weight, prenatal body mass index (BMI), prenatal body surface area, and parity at enrollment. Prenatal laboratory variables included platelet count, prothrombin time, thrombin time, urea, creatinine, uric acid, aspartate aminotransferase (AST), and alanine aminotransferase (ALT). All variables were collected within 1 week before delivery.

### 2.3. Immediate Postpartum Point-of-Care Ultrasound Assessment

A Mindray Z6 ultrasound diagnostic system (Mindray Bio-Medical Electronics Co., Ltd., Shenzhen, China) equipped with a 3C5P curvilinear probe (frequency 3.5–6.5 MHz) for lung ultrasound, and a 2P2P phased array probe (frequency 2–5 MHz) for echocardiography, were used. All 160 participants underwent standardized lung and cardiac ultrasound examinations during the immediate postpartum period, within 2 h after delivery. Ultrasound examinations were performed by an investigator with more than 3 years of experience in lung and cardiac ultrasound who was blinded to clinical information and pregnancy outcomes. Measurements were performed offline using stored images, and representative images were reviewed by a second experienced ultrasound physician when image quality or measurement boundaries were uncertain. The operator had more than 3 years of focused lung and cardiac ultrasound experience and had completed local competency training before study participation. Clinicians responsible for obstetric management were also blinded to ultrasound findings.

Lung ultrasound was performed using the 28-intercostal-space technique. The total number of B-lines across all scanning zones was recorded as the echo comet score (ECS), which was used as a semiquantitative marker of extravascular lung water and pulmonary congestion.

Cardiac ultrasound was performed according to recommendations from the American Society of Echocardiography and the European Association of Cardiovascular Imaging [29]. Parameters included left ventricular ejection fraction (LVEF), left atrial volume index (LAVI), left ventricular E/A ratio (LV E/A), mitral E-wave deceleration time (EDT), left ventricular index of myocardial performance (LIMP), right ventricular fractional area change (RVFAC), and inferior vena cava collapsibility index (IVC-CI).

A standardized immediate postpartum ultrasound protocol was followed for all participants, with details of the views and procedures provided in Supplementary Material (Table S1). The complete lung and cardiac ultrasound examination required approximately 10–15 min to perform for each participant.

### 2.4. Adverse Maternal Outcomes

Participants were followed for 42 days postpartum. The composite AMO included severe maternal complications involving neurologic, cardiopulmonary, hepatic, renal, and hematologic systems. These events were combined into a composite endpoint because they represent clinically meaningful maternal deterioration related to HDP and were selected based on severe maternal complications used in previous HDP outcome studies. Detailed outcome definitions are provided in the Supplementary Material (Table S2).

### 2.5. Statistical Analysis

Continuous variables were expressed as mean ± standard deviation or median (interquartile range), according to data distribution. Categorical variables were presented as counts and percentages. Between-group comparisons were performed using the independent-samples t-test or Mann–Whitney U-test for continuous variables and the χ^2^ test or Fisher’s exact test for categorical variables.

To evaluate the incremental value of immediate postpartum ultrasound variables for risk stratification, a baseline clinical model was constructed using conventional demographic, clinical, and laboratory variables. The baseline clinical model included gestational age, BMI, platelet count, thrombin time, urea, creatinine, uric acid, AST, ALT, and HDP subtype. HDP subtype was included because severe preeclampsia was more frequent among women with adverse maternal outcomes and was considered an important marker of disease severity. Given the limited number of outcome events, the model was intended for exploratory incremental assessment rather than development of a definitive clinical prediction tool. Ultrasound parameters were then added individually to the baseline model to evaluate their incremental value beyond conventional clinical assessment.

Model performance was assessed using the area under the receiver operating characteristic curve (AUC), Brier score, and Akaike Information Criterion (AIC). Improvements in model discrimination after incorporation of ultrasound variables were evaluated using the DeLong test. A two-sided *p* value < 0.05 was considered statistically significant. Statistical analyses were performed using R software (version 4.1.3; R Foundation for Statistical Computing, Vienna, Austria).

## 3. Results

### 3.1. Participant Characteristics

A total of 160 women with HDP were included in the final analysis, of whom 35 (21.88%) experienced AMO during the 42-day postpartum follow-up period. The incidence and distribution of each AMO are summarized in Table 1. The mean maternal age was 32.64 ± 4.39 years, 87 (54.38%) were nulliparous, and 122 (76.25%) underwent cesarean delivery.

Women who developed AMO had significantly lower gestational age at delivery and differed significantly in weight, BMI, and HDP subtype distribution compared with women without AMO. In addition, platelet count was significantly lower, whereas thrombin time, urea, creatinine, uric acid, AST, and ALT levels were significantly higher in the AMO group (all *p* < 0.05) (Table 2).

### 3.2. Immediate Postpartum Ultrasound Findings

**Figure 2:**
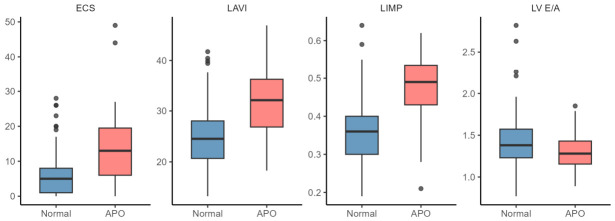
Distribution of principal ultrasound parameters by outcome status.

Significant differences in immediate postpartum lung and cardiac ultrasound findings were observed between women with and without AMO. Compared with women without AMO, those who experienced AMO demonstrated significantly higher ECS. In addition, LAVI and LIMP were significantly increased in the AMO group, while LV E/A was significantly reduced. No significant between-group differences were observed for LVEF, EDT, RVFAC, or IVC-CI (Table 3 and Figure 2).

### 3.3. Incremental Value of Ultrasound Parameters for Risk Stratification

The baseline clinical model incorporated gestational age, BMI, HDP subtype, platelet count, thrombin time, urea, creatinine, uric acid, AST, and ALT. This model yielded a Brier score of 0.11, an AIC of 134, and an AUC of 0.88 for identification of AMO.

**Figure 3:**
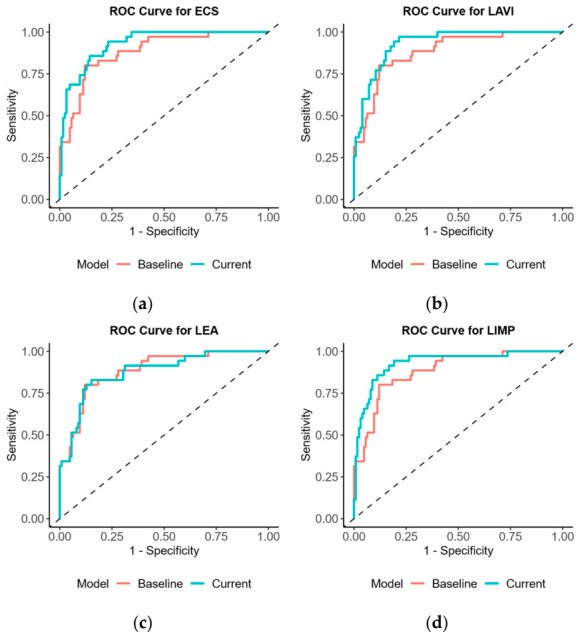
Receiver operating characteristic (ROC) curves showing the incremental discriminatory value of individual ultrasound parameters when added to the baseline clinical model for adverse maternal outcomes. (**a**) Echo comet score (ECS); (**b**) left atrial volume index (LAVI); (**c**) left ventricular E/A ratio (LEA); and (**d**) left ventricular index of myocardial performance (LIMP). The red line represents the baseline clinical model, and the blue line represents the model after addition of the corresponding ultrasound parameter.

When immediate postpartum ultrasound parameters were individually incorporated into the baseline model, ECS, LAVI, and LIMP each demonstrated incremental discriminatory value. Addition of these ultrasound-derived hemodynamic indicators improved model discrimination, with AUC values increasing to 0.93. Furthermore, models incorporating ECS, LAVI, or LIMP demonstrated lower Brier scores and lower AIC values compared with the baseline clinical model alone. In contrast, incorporation of LV E/A did not significantly improve discriminatory performance compared with the baseline model (Table 4 and Figure 3).

Additional analyses combining multiple ultrasound parameters showed consistent improvements in risk stratification performance, further supporting the complementary value of immediate postpartum maternal hemodynamic assessment (Supplementary Table S3 and Figure S1).

## 4. Discussion

### 4.1. Principal Findings

In this prospective cohort study of women with HDP, significant differences in immediate postpartum lung and cardiac ultrasound findings were observed between women with and without AMO. Specifically, women who developed AMO demonstrated higher ECS, LAVI, and LIMP values, suggesting increased pulmonary congestion and impaired maternal cardiovascular adaptation during the immediate postpartum period. Importantly, incorporation of these ultrasound-derived hemodynamic indicators provided incremental value beyond conventional clinical and laboratory variables for risk stratification of AMO.

### 4.2. Maternal Hemodynamic Adaptation and Ultrasound Findings

The intrapartum and immediate postpartum periods are characterized by profound cardiovascular and fluid shifts [9,10]. Uterine contractions, autotransfusion from the contracted uterus, and rapid increases in venous return substantially increase maternal preload and cardiac output [10,11]. In women with HDP, these physiologic stresses occur in the setting of endothelial dysfunction, increased vascular resistance, and impaired cardiac reserve, potentially predisposing patients to pulmonary congestion and cardiovascular decompensation [13,21].

Our findings support this physiologic framework. ECS, a semiquantitative marker of extravascular lung water, was significantly elevated in women with AMO, suggesting that subclinical pulmonary congestion may already be present during the immediate postpartum period in high-risk patients. Similarly, increased LAVI may reflect elevated left ventricular filling pressures and impaired diastolic adaptation, while elevated LIMP indicates combined systolic–diastolic myocardial dysfunction. Together, these findings indicate that ultrasound-derived markers capture clinically relevant aspects of maternal cardiopulmonary status associated with adverse outcomes and provide additional physiologic information beyond conventional clinical and laboratory variables. Nevertheless, these physiological interpretations remain speculative. Elevated ECS, LAVI, and LIMP may also serve as surrogate markers of overall HDP severity rather than representing independent pathophysiological mechanisms directly contributing to AMO.

### 4.3. Incremental Value Beyond Conventional Clinical Assessment

Several established prognostic models for HDP, including the fullPIERS and PREP models, primarily rely on demographic, clinical, and laboratory variables [25,26]. While these approaches provide useful estimates of disease severity, they do not directly evaluate maternal cardiovascular adaptation during labor and the early postpartum period.

In the present study, addition of ECS, LAVI, or LIMP to the baseline clinical model improved discriminatory performance and overall model fit. These findings suggest that immediate postpartum ultrasound-derived hemodynamic information may complement conventional risk assessment by providing real-time physiologic insight into maternal cardiopulmonary status. Importantly, the observed improvement was achieved using rapid bedside ultrasound techniques that are noninvasive and widely accessible in contemporary obstetric practice. Given the observational design of this study, these findings indicate associations and incremental discriminatory value rather than causal relationships between abnormal ultrasound parameters and adverse maternal outcomes. Although HDP subtype was included in the baseline clinical model, residual confounding by disease severity cannot be excluded. Therefore, the incremental discriminatory value of ECS, LAVI, and LIMP should not be interpreted as evidence that these parameters provide prognostic information entirely independent of HDP severity.

Rather than serving as a replacement for conventional clinical assessment, immediate postpartum POCUS may function as an adjunctive bedside tool for identifying women at increased risk of postpartum deterioration. This may be particularly relevant in women with severe preeclampsia or evidence of fluid redistribution, in whom timely recognition of pulmonary congestion or impaired cardiac adaptation could influence monitoring intensity and postpartum management strategies.

Our findings are also consistent with emerging evidence from the field of cardio-obstetrics demonstrating that women with HDP frequently exhibit subclinical cardiac remodeling, impaired diastolic function, and elevated filling pressures even in the absence of overt cardiovascular symptoms [30,31]. These observations further support the concept that ultrasound-derived hemodynamic assessment may provide clinically relevant information beyond conventional obstetric evaluation.

### 4.4. Clinical Implications

The clinical significance of our findings lies in the potential role of immediate postpartum ultrasound in maternal hemodynamic monitoring and early postpartum risk stratification. Conventional obstetric assessment may not fully reflect the dynamic cardiovascular changes occurring during labor and immediately after delivery. By contrast, bedside lung and cardiac ultrasound can provide rapid visualization of pulmonary fluid status and cardiac functional adaptation in real time. In addition, POCUS utilizes standard ultrasound equipment that is already available in most obstetric units and can be completed within approximately 10–15 min, making it a relatively low-cost and feasible bedside assessment tool. Although adequate operator training is required, both lung ultrasound and focused cardiac ultrasound can be incorporated into routine obstetric practice following standardized training programs and competency assessment. As POCUS becomes increasingly integrated into obstetric critical care and cardio-obstetrics practice, incorporation of focused maternal hemodynamic assessment into existing obstetric workflows may help identify women with HDP who require closer postpartum surveillance or more intensive cardiovascular monitoring. Further multicenter studies are needed to determine whether ultrasound-guided monitoring strategies can improve maternal outcomes. And future studies with larger cohorts are needed to establish clinically meaningful threshold values for ECS, LAVI, and LIMP that may facilitate bedside risk stratification. The present study was designed to evaluate the incremental risk-stratification value of ultrasound-derived parameters rather than to establish diagnostic or prognostic cutoff values. Given the modest number of adverse maternal outcome events and the absence of an independent validation cohort, data-driven thresholds derived from the present dataset may be unstable and overly optimistic. Larger prospective studies with independent validation cohorts are needed to determine clinically applicable thresholds for ECS, LAVI, and LIMP.

### 4.5. Strengths and Limitations

This study has several strengths. First, its prospective design reduced recall bias and ensured standardized ultrasound acquisition. Second, all ultrasound examinations were performed within a clinically relevant time window characterized by maximal maternal hemodynamic stress. Third, clinicians responsible for patient management were blinded to ultrasound findings, thereby minimizing intervention bias.

Several limitations should also be acknowledged. The study was conducted at a single tertiary referral center in China, and participant recruitment was non-consecutive because only daytime deliveries were enrolled. This may have introduced selection bias, and the enrolled cohort may not fully represent all women with HDP treated during the study period. The number of adverse maternal outcome events was modest, creating a risk of model overfitting and unstable coefficient estimates; therefore, the baseline and ultrasound-enhanced models should be regarded as exploratory. Severe preeclampsia was overrepresented among women with adverse maternal outcomes, and ultrasound abnormalities may partly reflect disease severity rather than independent prognostic pathways. External validation in larger, multicenter cohorts is required before clinical implementation.

## 5. Conclusions

In women with HDP, immediate postpartum lung and cardiac ultrasound findings differed significantly according to postpartum outcome status. Ultrasound-derived maternal hemodynamic indicators, particularly ECS, LAVI, and LIMP, provided incremental value beyond conventional clinical variables for risk stratification of adverse maternal outcomes. Immediate postpartum POCUS may therefore represent a useful adjunctive tool for bedside maternal cardiovascular assessment during the high-risk peripartum period. However, given the single-center design, modest sample size, non-consecutive enrollment, and lack of external validation, larger multicenter studies are warranted to confirm these findings and further establish their clinical applicability.

## Figures and Tables

**Table 1:** Adverse maternal outcomes included in this study.

AMO	Number
Maternal death	
Death occurring at any time from delivery to hospital discharge	0
Central Nervous System	
Reversible ischemic neurological deficit (24–48 h)	1 (2.86%)
Cardiopulmonary System	
Oxygen therapy > 50% concentration > 1 h	1 (2.86%)
Pulmonary edema (X-ray or clinical, SaO_2_ < 94%)	5 (14.29%)
Hepatic System	
DIC-suggestive coagulopathy (INR > 1.2 in the absence of Warfarin)	1 (2.86%)
Elevated transaminase > 2 times the upper limit of normal	9 (25.71%)
Renal System	
Acute kidney injury (per KDIGO criteria)	12 (34.29%)
Hematological System	
Transfusion of any blood product	11 (31.43%)
Thrombocytopenia (platelet count < 100 × 109/L)	16 (45.71%)
Postpartum hemorrhage (>1 L)	3 (8.57%)

Outcome components were not mutually exclusive; therefore, one participant could contribute to more than one component. Percentages were calculated using the 35 women with AMO as the denominator. AMO, adverse maternal outcome. SaO_2_, arterial oxygen saturation. DIC, disseminated intravascular coagulation. INR, international normalized ratio. KDIGO, Kidney Disease: Improving Global Outcomes.

**Table 2:** Demographic and laboratory test of AMO compared to normal outcomes.

Characteristic	AMO (n = 35)	Normal (n = 125)	*p*
Demographic			
Maternal age, years	33.26 ± 3.68	32.46 ± 4.56	0.290
Gestational age, days	258 (245.5–266.5)	267 (261–273)	<0.001 ^§^
Height, cm	160 ± 5.08	159.05 ± 5.25	0.335
Weight, kg	73.15 ± 13	78.25 ± 11.56	0.041
BMI, kg/m^2^	28.76 (25.13–31.25)	30.04 (28.19–32.79)	0.005 ^§^
BSA, m^2^	1.76 ± 0.18	1.82 ± 0.17	0.090
Nulliparous	19 (54.29%)	68 (54.40%)	1.000†
Type			0.002†
Gestational hypertension	6 (17.14%)	37 (29.60%)	0.002 ^†^
PE	6 (17.14%)	42 (33.60%)	
PE with severe features	16 (45.71%)	18 (14.40%)	
Eclampsia	0	0	
Pregnancy complicated with chronic hypertension	0	7 (5.60%)	
PE superimposed upon chronic hypertension	7 (20.00%)	21 (16.80%)	
Laboratory test			
Platelet count, 10^9^/L	185.57 ± 72.76	238.46 ± 61.29	<0.001
Prothrombin time, s	10.3 ± 0.82	10.5 ± 0.89	0.209
Thrombin time, s	14.5 (13.55–15.5)	13.5 (12.6–15.3)	0.023 ^§^
Urea, mmol/L	5.67 ± 2.41	4.06 ± 1.48	<0.001
Creatinine, μmol/L	59.73 ± 19.19	50.43 ± 10.71	0.009
Uric acid, μmol/L	455.83 ± 102.75	387.21 ± 85.6	<0.001
AST, U/L	24.1 (17.95–28.5)	18 (15.7–21.9)	0.001 ^§^
ALT, U/L	16.6 (12.25–23.95)	10.9 (8.1–14.2)	<0.001 ^§^

Data are given as mean ± SD, median (interquartile range), n (%). ^§^ Mann–Whitney U-test. ^†^ Chi-square test or Fisher’s exact test. AMO, adverse maternal outcomes. BMI, body mass index. BSA, body surface area. PE, preeclampsia. AST, aspartate aminotransferase. ALT, alanine transaminase.

**Table 3:** Immediate postpartum ultrasound variables of AMO compared to normal outcomes.

Ultrasound Variables	AMO (n = 35)	Normal (n = 125)	95% CI	*p*
ECS	13 (6–19.5)	5 (1–8)	[−14, −4]	<0.001 ^§^
LVEF, %	66.17 ± 5.13	66.47 ± 5.51	[−1.58, 2.27]	0.759
LAVI, cm^2^/m^2^	32.13 ± 6.82	25.07 ± 6.43	[−9.51, −4.61]	<0.001
LV E/A	1.3 ± 0.24	1.43 ± 0.31	[0.04, 0.23]	0.009
EDT, ms	194.57 ± 37.68	191.76 ± 35.54	[−16.2, 10.71]	0.694
LIMP	0.48 ± 0.09	0.36 ± 0.08	[−0.16, −0.09]	<0.001
RVFAC, %	47.67 ± 6.61	47.78 ± 6.67	[−2.31, 2.63]	0.927
IVC-CI, %	25.87 ± 10.74	26.05 ± 10.63	[−3.83, 3.93]	0.928

Data are given as mean ± SD, median (interquartile range). ^§^ Mann–Whitney U-test. AMO, adverse maternal outcomes. ECS, echo comet score. LVEF, left ventricle ejection fraction. LAVI, left atrial volume index. LV E/A, left ventricular E/A ratio. EDT, e-peak deceleration time. LIMP, left ventricular index of myocardial performance. RVFAC, right ventricle fractional area change. IVC-CI, inferior vena cava collapsibility index.

**Table 4:** Incremental value of immediate postpartum ultrasound parameters for risk stratification of adverse maternal outcomes.

Model	Brier	AIC	AUC (95% CI)	DeLong’s *p*
Baseline	0.11	134	0.88 (0.82–0.94)	NA
Baseline + ECS	0.09	116	0.93 (0.89–0.97)	0.022
Baseline + LAVI	0.09	116	0.93 (0.89–0.97)	0.039
Baseline + LEA	0.11	135	0.88 (0.81–0.94)	0.446
Baseline + LIMP	0.08	113	0.93 (0.88–0.98)	0.045

AIC, Akaike information criterion. AUC, area under curve. ECS, echo comet score. LAVI, left atrial volume index. LV E/A, left ventricular E/A ratio. LIMP, left ventricular index of myocardial performance.

## Data Availability

The datasets generated and/or analyzed during the current study are not publicly available due to privacy and ethical restrictions.

## Supplementary Materials

The following supporting information can be downloaded at: https://www.mdpi.com/article/10.3390/jcm15134989/s1, Table S1: Standardized ultrasound protocol; Table S2: Adverse maternal outcomes; Table S3: Analyses combining multiple ultrasound parameters; Figure S1: ROC curve of analyses combining multiple ultrasound parameters.

## Abbreviations

The following abbreviations are used in this manuscript:

ACOG	American College of Obstetricians and Gynecologists
AIC	Akaike Information Criterion
ALT	Alanine Aminotransferase
AMO	Adverse Maternal Outcomes
AST	Aspartate Aminotransferase
AUC	Area Under the Receiver Operating Characteristic Curve
BMI	Body Mass Index
ECS	Echo Comet Score
EDT	E-wave Deceleration Time
HDP	Hypertensive Disorders of Pregnancy
IVC-CI	Inferior Vena Cava Collapsibility Index
LAVI	Left Atrial Volume Index
LIMP	Left Ventricular Index of Myocardial Performance
LV E/A	Left Ventricular E/A Ratio
LVEF	Left Ventricular Ejection Fraction
POCUS	Point-of-Care Ultrasound
RVFAC	Right Ventricular Fractional Area Change
