# Supplementary material for: Incremental Value of Immediate Postpartum POCUS for Risk Stratification of Adverse Maternal Outcomes in Hypertensive Disorders of Pregnancy
Source: J Clin Med. 2026 Jun 26;15(13):4989. doi: 10.3390/jcm15134989 (PMC13361736; doi:10.3390/jcm15134989)

Table S1 Standardized ultrasound protocol

| Ultrasound variable | View and method | Measurement description and key points |
|---|---|---|
| ECS | 28-intercostal scanning protocol | Scanned at left 2nd–4th and right 2nd–5th intercostal spaces along mid-axillary, anterior axillary, midclavicular, and parasternal lines. The total number of B-lines across 28 sites was summed to obtain the ECS. |
| LVEF | Apical four- and two-chamber views | Measured by the biplane Simpson's method. End-diastolic volume (EDV) and end-systolic volume (ESV) were measured by tracing the endocardial border of the left ventricle, with closure at the mitral annulus level. Formula: LVEF = (EDV − ESV) / EDV × 100%. |
| LAVI | Apical four- and two-chamber views | 2D volumetric measurements are based on tracings of the blood-tissue interface on apical four- and two-chamber views. At the mitral valve level, the contour is closed by connecting the two opposite sections of the mitral annulus with a straight line. Endocardial tracing should exclude atrial appendage and pulmonary veins. Left atrial length (L) is defined as the shortest of the two long axes measured in the apical two- and four-chamber views (the two lengths should not differ by more than 5 mm). Left atrial volume (LAV) can be computed using the area–length approximation: LAV = 8/(3π) × (A1 × A2 / L), where A1 and A2 are the corresponding left atrial areas. LAVI is calculated by dividing LAV by body surface area. |
| LV E/A ratio | Apical four-chamber view | Pulsed Doppler sample volume placed at mitral leaflet tips. Recorded early (E) and late (A) diastolic velocities to calculate E/A ratio. |
| EDT | Apical four-chamber view | E-wave deceleration time: interval from E-wave peak to extrapolated baseline. |
| LIMP | Apical four- and five-chamber views | Measurements were obtained using pulsed-wave Doppler. First, mitral inflow spectra (E and A waves) were recorded from the apical four-chamber view. The probe was then tilted slightly superiorly and toward the right shoulder to obtain the apical five-chamber view, showing the left ventricular outflow tract and aortic valve. The sample volume was placed at the aortic valve tip to record the aortic outflow velocity. Heart rate was maintained relatively stable during both recordings. Formula: LIMP=(TCO-ET)/ET, where TCO represents the interval between mitral valve closure and reopening (measured from the end of the A-wave to the onset of the next E-wave), and ET is the ejection time measured from the aortic flow velocity spectrum. |
| RVFAC | Apical four-chamber view with right ventricle as the main component | The endocardial border of the right ventricle was manually traced from the lateral tricuspid annulus along the free wall to the apex and back along the septum, both at end-diastole (EDA) and end-systole (ESA). Formula: RVFAC = (EDA − ESA) / EDA × 100%. |
| IVC-CI | Subxiphoid or subcostal long-axis view | Using a subxiphoid or subcostal long-axis view, the IVC was visualized approximately 0.5–1.0 cm proximal to the junction with the hepatic veins and right atrium. The end-expiratory (IVCe) and end-inspiratory (IVCi) diameters were measured perpendicular to the vessel axis. Formula: IVC-CI=(IVCe-IVCi) / IVCe x 100%. |

Table S2 Adverse maternal outcomes

| Based on previous studies 25, 26, we included the following maternal outcomes: |
| --- |
| (1) Maternal Death: Maternal death occurring at any time from delivery to hospital discharge. |
| (2) Central Nervous System: Glasgow Coma Scale score <13. Stroke (acute neurological event lasting more than 48 hours). Cortical blindness (loss of vision with normal pupillary light reflexes). Retinal detachment. Reversible ischemic neurological deficit (cerebral ischemia identified by clinical examination, lasting more than 24 hours but less than 48 hours). |
| (3) Cardiopulmonary System: Requirement of oxygen therapy >50% concentration for more than 1 hour. Intubation not related to cesarean delivery (possibly for ventilation, electrical impedance tomography, or continuous positive airway pressure). Pulmonary edema (confirmed by X-ray or clinical diagnosis requiring diuretics with SaO2 <94%). Vasopressor support (use of vasopressors to maintain systolic blood pressure> 90 mmHg or mean arterial pressure >70 mmHg). Myocardial ischemia/infarction (ischemic changes on electrocardiogram, possibly accompanied by pathological Q waves or coronary intervention). |
| (4) Hepatic System: Liver dysfunction (INR >1.2, suggesting disseminated intravascular coagulation). Transaminase levels >2 times the upper limit of normal. Hepatic hematoma or rupture (subcapsular hematoma confirmed by ultrasound or laparotomy). |
| (5) Renal System: Acute kidney injury (increase in serum creatinine ≥26 μ mol/L within 48 hours, >50% increase in serum creatinine over the past 7 days, urine output <0.5 mL/kg/hr for >6 hours, or serum creatinine >150μmol/L in the absence of baseline serum creatinine values). Dialysis (hemodialysis or peritoneal dialysis). |
| (6) Hematological System: Blood transfusion (including fresh frozen plasma, platelets, red blood cells, cryoprecipitate, or whole blood). Thrombocytopenia (platelet count <100×109/L). Postpartum hemorrhage (blood loss >1 L following delivery). |

1. von Dadelszen P, Payne B, Li J, Ansermino JM, Broughton Pipkin F, Côté AM, et al. Prediction of adverse maternal outcomes in pre-eclampsia: development and validation of the fullPIERS model. Lancet. 2011;377(9761):219-227.

2. Thangaratinam S, Allotey J, Marlin N, Dodds J, Cheong-See F, von Dadelszen P, et al. Prediction of complications in early-onset pre-eclampsia (PREP): development and external multinational validation of prognostic models. BMC medicine. 2017;15(1):68.

Table S3 Analyses combining multiple ultrasound parameters

| model | Brier | AIC | AUC (95%CI) | DeLong's P |
|---|---|---|---|---|
| Baseline | 0.11 | 134 | 0.88 (0.82-0.94) | NA |
| ECS+LAVI | 0.08 | 107 | 0.95 (0.92-0.98) | 0.002 |
| ECS+LIMP | 0.08 | 108 | 0.94 (0.90-0.98) | 0.005 |
| LAVI+LIMP | 0.07 | 103 | 0.95 (0.92-0.98) | <0.001 |
| ECS+LAVI+LIMP | 0.07 | 101 | 0.96 (0.92-0.98) | <0.001 |

Figure S1 ROC curve of analyses combining multiple ultrasound parameters

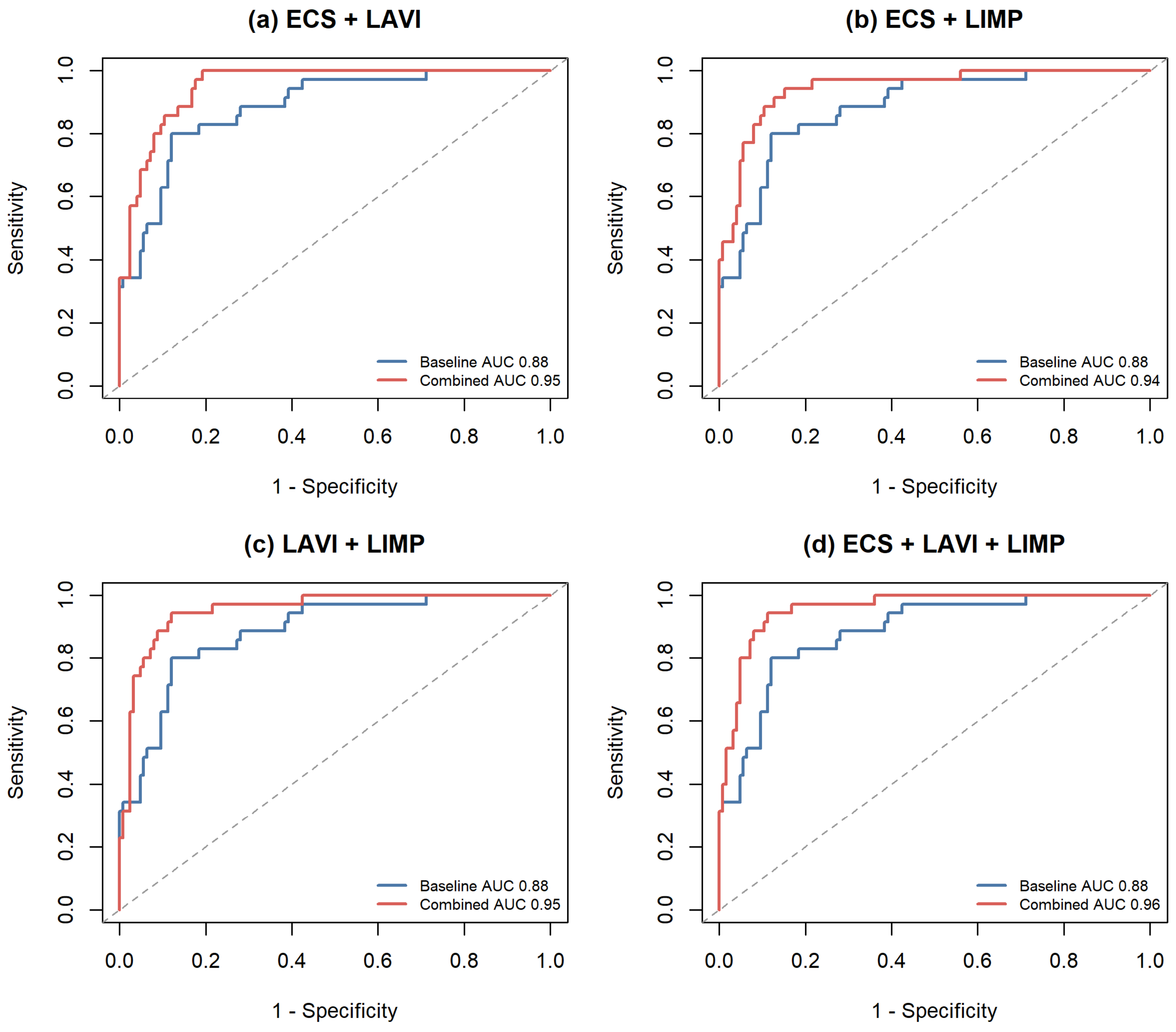

Supplement: Supplementary file 1 [file jcm-15-04989-s001.zip › jcm-4381699-supplementary.pdf]
